# Supplementary material for: Digital Mental Health Interventions for Young People in Rural South Africa: Prospects and Challenges for Implementation
Source: Int J Environ Res Public Health. 2023 Jan 13;20(2):1453. doi: 10.3390/ijerph20021453 (PMC9859354; doi:10.3390/ijerph20021453)
Supplement: Supplementary file 1 [file ijerph-20-01453-s001.zip › ijerph-2065977-supplementary.pdf]

Latest EMIDIYA questionnaire 2020

ngitsheliwe kabanzi ngokuthi lolucwaningo lungani. Ngiyaqonda inhloso nezinqubo zalolucwaningo. Nginikezwe ithuba lokuphendula imibuzo emayelana nalolucwaningo futhi ngiphenduliwe ngagculiseka. Ngiyavuma ukuthi ukuzibandakanya kwami kulolu cwaningo kungokuzithandela ngokuphelele nokuthi ngingahoxa nganoma yisiphi isikhathi ngaphandle kolahlekelwa noma yiziphi izinzuzo engivame ukuba nazo. Ngichazeliwe nganoma yisiphi isinxephezelo esitholakalayo noma izindlela zokwelashwa uma kwenzeka ngilimala ngenxa yezinqubo ezihlobene nalolucwaningo. Uma ngineminye imibuzo/ izikhalo noma izicelo ezihlanganisa nalolucwaningo, ngiqonda ukuthi ngingaxhumana nomcwaningi. Uma nginemibuzo noma izikhalo ngamalungelo ami njengombambiqhaza kulolucwaningo, noma uma nginesikhalo ngenxenye yalolucwaningo noma ngomcwaningi ngingaxhumana nalaba abalandelayo: HUMANITIES & SOCIAL SCIENCES RESEARCH ETHICS ADMINISTRATION Research Office, Westville Campus Govan Mbeki Building Private Bag X 54001 Durban 4000 KwaZulu-Natal, SOUTH AFRICA Tel: 27 31 2604557 - Fax: 27 31 2604609 Email: HSSREC@ukzn.ac.za

Signature

Name of participant

____

Age

____

Sex

Ubulili

- ◯ Owesifazane
- ◯ Owesilisa

Level of education

- ☐ Matric
- ☐ College
- ☐ Primary school

1. Iziphi izinkinga ezijwayelekile ezihlanganise nempilo yenqondo ezithinta intsha emphakathini wenu?

- [ ] Ukusetshenziswa kabi kwezidakamizwa
- [ ] ukucindezeleka
- [ ] udlame
- [ ] ukuhlukumezeka
- [ ] Okunye

Add Okunye

____

2. Intsha ikufuna kuphi/kanjani ukwelashwa noma usizo uma inenkinga ehlanganisa nempilo yenqondo?

- [ ] Umtholampilo wenqondo/isibhedlela
- [ ] umsebenzi wezempilo womphakathi
- [ ] Sangoma
- [ ] Olapha ngokomoya
- [ ] Indlu yesonto
- [ ] Okunye

Add okunye

____

3. Intsha idinga ziphi izinsizakalo zempilo yenqondo?

- [ ] Ukwelulekwa
- [ ] Ukulashwa
- [ ] Ukwelashwa
- [ ] Ukuvuselelwa
- [ ] Okunye

Okunye

____

4. Chaza ukuthi yini oyaziyo ngokuhlukumezeka kwenqondo.

____

5. Zitholakala kuphi izinsizakalo zempilo yenqondo?

____

6. Ngabe intsha yalomphakathi iyaba nezinkinga zokuhlukumezeka kwenqondo?

- ◯ Cha
- ◯ Yebo

7. Kulomphakathi iziphi izimbangela ezivamile zokuhlukumezeka kwenqondo kwintsha?

____

8. Ngokucabanga kwakho izidakamizwa ziyayidlala yini indima enkulu ekuhlukemezekeni kwenqondo yentsha kulomphakathi?

- ◯ Cha
- ◯ Yebo

9. Ucabanga ukuthi ukusebenzisa usizo oludijithali lwempilo yenqondo njengenkundla yokuxwayisa entsheni lungasiza?

- ◯ Yebo
- ◯ Cha

10. Zikhona izinsiza ezidijithali zempilo yenqondo ezikhona ezitholakala uma umuntu enencindezi noma ephazamiseka ngokwenqondo?

- ◯ Cha
- ◯ Yebo

Yiziphi lezo izinsiza

____

11. Abacwaningi/ abasebenzi bezempilo sebake bafika kini nezmibuzo emayelana nempilo yenqondo?

- ◯ Yebo
- ◯ Cha

12. Ucabanga ukuthi ukusebenzisa amathuluzi eselula okuzihlola kungasiza ukuthi intsha iqonde impilo yenqondo?

- ◯ Yebo
- ◯ Cha

13. Ingabe kulungile ukuba intsha ithole usizo lwempilo yenqondo kwi-inthanethi?

- ◯ Yebo
- ◯ Cha

14. Iziphi izinkinga intsha engahlangabezana nazo uma isebenzisa i-inthanethi?

____

15. Uhlobo luni lwedivayisi oyisebenzisayo uma ungena kwi-inthanethi

- [ ] Phone
- [ ] Tablet
- [ ] laptop
- [ ] Computer

16. Iziphi izinkundla zokuxhumana ongaqoka ukuthi zikufundise ngezinkinga zempilo yenqondo nanokuthi siphathwa kanjani lesosimo

____

17. Yini oyithandayo nongayithandi ngezinkundla zokuxhumana ku intanethi?

____

18. Ungazichaza kanjani njengzomsebenzisi we-inthanethi?

- ( ) Ngiyisebenzisa kakhulu i-inthanethi
- ( ) Ngiyisebenzisa kancaane i-inthanethi

19. Ekuqaleni, yini eyanikhuthaza ukuba nikhethe lenkundla yokuxhumana eniyisebenzisayo njengamanje?

____

18. Ngokwazi kwenu njengabantu abasebenzisa i-inthanethi, izinkundla zokuxhumana zibaluleke kangakanani?

- ( ) Zibaluleke kakhulu
- ( ) Zibaluleke kancane

19. Nizisebenzisa kangakanani izinkundla zokusebenza/zokuxhumana?

- [ ] Ngaso sonke isikhathi
- [ ] Kancane nje

20. Nizisebenzisela ini okunye ngaphandle kokuxhumana nabanye?

____

21. Iyiphi indlela ongayiqoka encono kuwe ukuba uxhumane nomsebenzi wezempilo yenqondo?

- [ ] i. Ngohlelo lokusebenza lweselula, ocingweni noma ukuthumela imiyalezo
- [ ] ii. Ngezinkundla zokuxhumana: ku-Facebook, ku-Twitter,ku- Whatsapp, ku-Instagram, Okunye
- [ ] iii. Ngengxoxo ekuwebsite, ngenkomfa yevidiyo

22. Uma usebenzisa uhlelo lomsebenzi ukuxhumana nomsebenzi wezempilo, hlobo luni lwezici ongazifuna zibe khona kuloluhlelo lokusebenza.

- [ ] a. Ukuvunyelwa ukuzifihla
- [ ] b. Ukwaziwa igama nobuso
- [ ] c. Imiyalezo yezwi
- [ ] d. Ukufonelana
- [ ] e. Ukufonelana nibonane
- [ ] f. Inkomfa yevidiyo
- [ ] g. Ukwabelana kwendawo umuntu akuyo
- [ ] h. Izici zokuthumela imiyalezo
- [ ] i. Ukusebenzisa ama emoji
- [ ] j. AmaGIFs akhombisa imizwelo
- [ ] k. Ukwabelana kwezithombe
- [ ] l. Ukwabelana kwama vidiyo

Okunye:

____

23. Usuwake wafonelwa umsebenzi wempilo ocingweni?

- ( ) Yebo
- ( ) Cha

24. Usuwake wafundiswa ngempilo yenqondo?

- ( ) Yebo
- ( ) Cha

25. Wayithola kanjani lemfundiso?

____

26. Sicela ukwazi ukuthi ungafuna umyalezo ufike ngandlelani kuwena

- [ ] i. Ngewebsite
- [ ] ii. Ngama mobile app
- [ ] iii. Nge- social media
- [ ] iv. Ngama voice or text massages
- [ ] v. Ngama Vidiyo
- [ ] vi. Ngama audio media

27. Kubalulekile ukuthi intsha ikwazi ukusebenzisa inkundla yokusebenza edijithali yokusebenza lwempilo yenqondo ukuba bakwazi ukuhlola izinkinga abangase babenazo zokuhlukumezeka inqondo

- Yebo
- Cha

28. Ubona ngathi umthwalo wakho ukuzihlola izinkinga zokuhlukumezeka enqondweni kulomphakathi ngokusebenzisa i-mobile app?

- Yebo
- Cha

29. Kuyakuphoqa yini ukuthi ufune usizo lochwepheshe bempilo yenqondo ngezinkinga eziphathelene nokuhlukumezeka kwenqondo?

- Yebo
- Cha

30. Unawo amakhono anele okusebenzisa ama Apps empilo yenqondo edijithali ukuze ukwazi ukuzihlola?

- Yebo
- Cha

31. Ngabe kumosha iskhathi ukufundisa intsha ngokuzihlola kwi-mobile app ukukhomba izinkinga zempilo yenqondo?

- Yebo
- Cha

32. Ama-Workshops edigital mental health angaba wusizo Phakathi kwentsha kulomphakathi na?

- Yebo
- Cha

33. Sikhona isidingo sokuba nenkundla yokuxhumana ebhekene nezinkinga zempilo yenqondo ngo-Facebook, u-twitter, u-WhatsApp, u-Instagram kanye ne-mobile app?

- Yebo
- Cha

34. Yini okumele icatshangwe uma intsha yaseAfrica yenzelwa izinhlelo zokusebenza ocingweni (Apps)?

____

35. Iziphi izithiko zamasiko engahlangabezana nazo intsha yaseAfrica esebenzisa lenhlelo yomsebenzi yocingo?

____
